# A Case Report of Rare Synchronous Esophageal Malignancies With Dissimilar Histology: Squamous Cell Carcinoma and Small Cell Carcinoma

**DOI:** 10.7759/cureus.29645

**Published:** 2022-09-27

**Authors:** Rutwik P Sharma, Amol Harshe, Pranjali Sharma, Pradeep P Sharma

**Affiliations:** 1 Internal Medicine, Rochester Regional Health, Rochester, USA; 2 Pathology, Noble Hospitals & Research Centre, Pune, IND; 3 Endocrinology, Parkview Medical Center, Pueblo, USA; 4 Surgery, Jehangir Hospital and Medical Centre, Pune, IND

**Keywords:** esophagus, small cell carcinoma, neuroendocrine tumor (net), synchronous malignancies, synchronous cancers, squamous carcinoma

## Abstract

The presence of synchronous primary malignancies is a rare phenomenon reported in the literature. Most synchronous malignancies reported include carcinomas and adenocarcinomas of the gastrointestinal tract, head and neck cancers, thyroid and breast cancers. Among the neuroendocrine tumors, carcinoid tumors in the duodenum or the esophagus are most commonly reported with other primary malignancies. We report the case of a 56-year-old male with tobacco use disorder, presenting with dysphagia and weight loss for six months, who was thought to have multicentric squamous cell carcinoma of the esophagus. In actuality, he was diagnosed with synchronous metastatic neuroendocrine tumor (NET) favoring small cell carcinoma and squamous cell carcinoma of the esophagus. The patient responded well to minimally invasive thoracoscopic esophagectomy with regional lymphadenectomy followed by chemotherapy and radiation therapy. We have not been able to find a literature referencing the presence of synchronous small cell carcinoma and squamous cell carcinoma of the esophagus, making our case unique.

## Introduction

The incidence of multiple primary malignancies has been studied as far back as the 1920s [1-3]. While a 1921 study of 3000 cases suggested an incidence of 4.7%, more recent epidemiological data shows that the frequency of multiple primary malignancies ranges between 2-17% [4].

Esophageal cancer is the third most common cancer in India [5] and eighth most common cancer in the world as per World Cancer Research Fund. The incidence of multiple primary cancers in patients with esophageal cancers has been reported to be between 5% and 36% [6]. Squamous cell carcinoma of the esophagus is more commonly associated with a second primary cancer in the oral cavity and pharynx, larynx, and lung [7]. A second primary cancer in the esophagus occurring synchronously with squamous cell carcinoma of the esophagus is rare, particularly in real-world practice.

We describe a rare case of two synchronous esophageal malignancies with dissimilar histology - squamous cell carcinoma and neuroendocrine tumor (NET) favoring small cell carcinoma - in two different locations of the esophagus.

## Case presentation

A 56-year-old male with a history of tobacco use disorder, presented with progressive dysphagia for six months. Dysphagia was more prominent with solid foods than liquids. He also reported a weight loss of 17.6 lbs (8 kg) over the same period. On examination, the patient was in no acute distress. There was no supraclavicular lymphadenopathy or organomegaly on abdominal examination. His complete blood count and metabolic panel were within normal range.

A barium swallow study showed mucosal irregularity in the upper and lower third of the esophagus. Computed tomography (CT) scan with contrast revealed a small tumor at 8 cm and a larger tumor at 20 cm from the hypopharynx. The patient then underwent an esophagogastroscopy and both lesions were biopsied. The histopathology of the upper tumor showed dysplastic cells, while that of the lower tumor revealed well-differentiated squamous cell carcinoma. Positron emission tomography (PET) CT scan did not reveal systemic spread suggesting no distant metastasis. At this point, the patient was thought to have multicentric squamous cell carcinoma of the esophagus.

**Figure 1:**
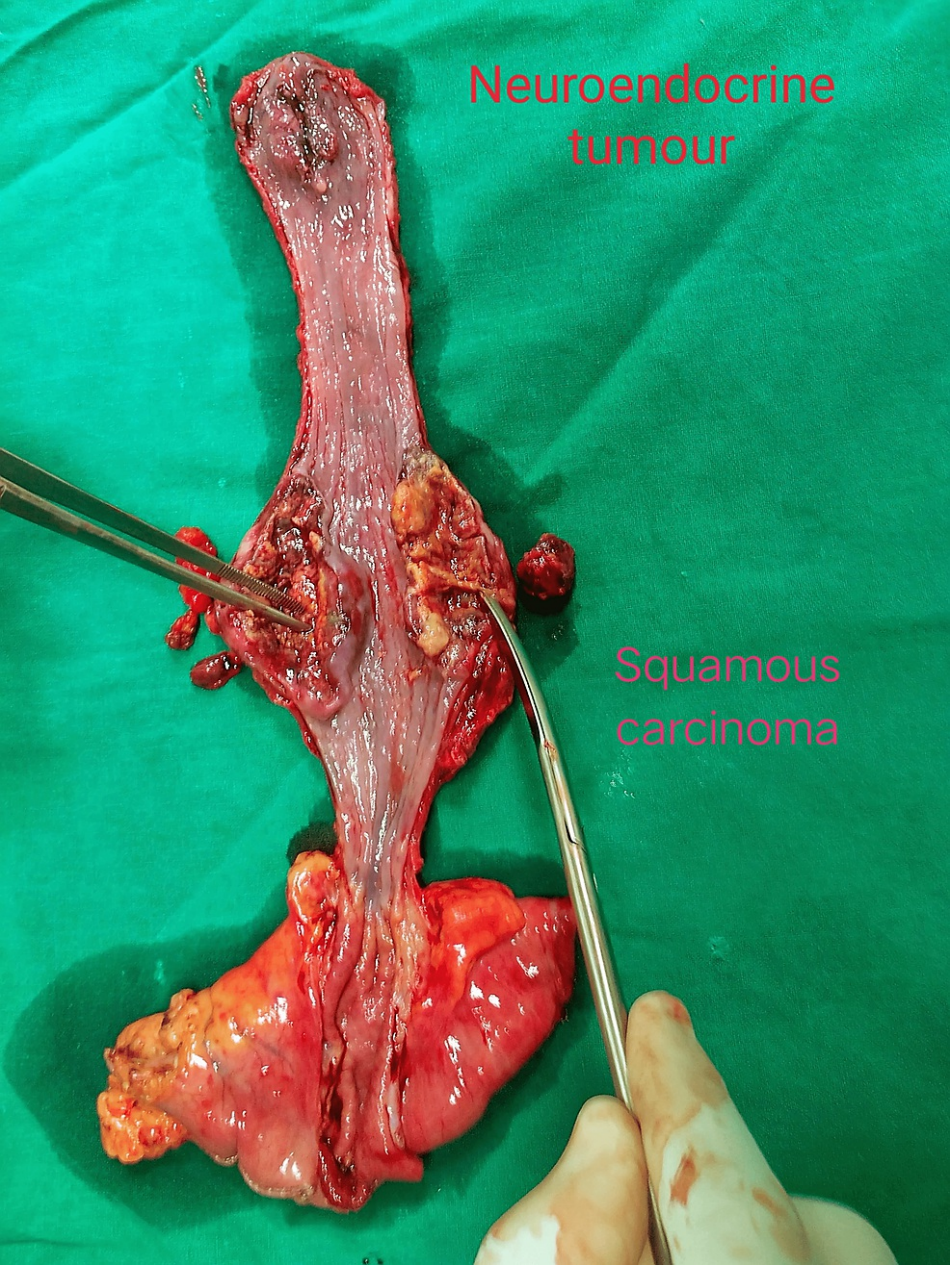
Specimen of Esophagus and Upper Stomach Removed in Continuity

After a discussion at the hospital's multicentric tumor board meeting, surgical resection of the esophagus was recommended. Due to preserved general condition of the patient, a minimally invasive approach to resection was chosen. The patient consented for a thoracoscopic esophagectomy with two-field regional lymphadenectomy, using the three-port method and prone position. The neck was opened through a left oblique incision. The esophagus was encircled and transected in the neck. Simultaneously an upper midline laparotomy was performed. The stomach was mobilized in a standard fashion and a proximal upper partial gastrectomy was done based on the lesser curvature. The specimen of the esophagus and upper stomach was removed in continuity (Figure 1). On the gross specimen, the upper tumor was 2 cm in length while the lower tumor was 5 cm in length (Figure 1).

A gastric tube was prepared along the greater curvature of the stomach. A margin of 5 cm could be achieved at the proximal end and 15 cm distally. The gastric tube was sutured to a feeding tube passed down from the neck. An end-to-end anastomosis was performed in a single layer with 3-0 Polyglycolic acid inverted sutures. The neck, esophageal bed, and abdomen were drained appropriately. A feeding jejunostomy was then added.

The patient made an uneventful recovery. The final histopathology report showed a grade 2 moderately differentiated squamous cell carcinoma in the lower third esophagus, 2.5 x 1.8 x 0.9 cm in dimensions, extending up to the serosa, and without lymphovascular emboli or perineural invasion. The circumferential resectional margin (CRM) was free of tumor.

**Figure 2:**
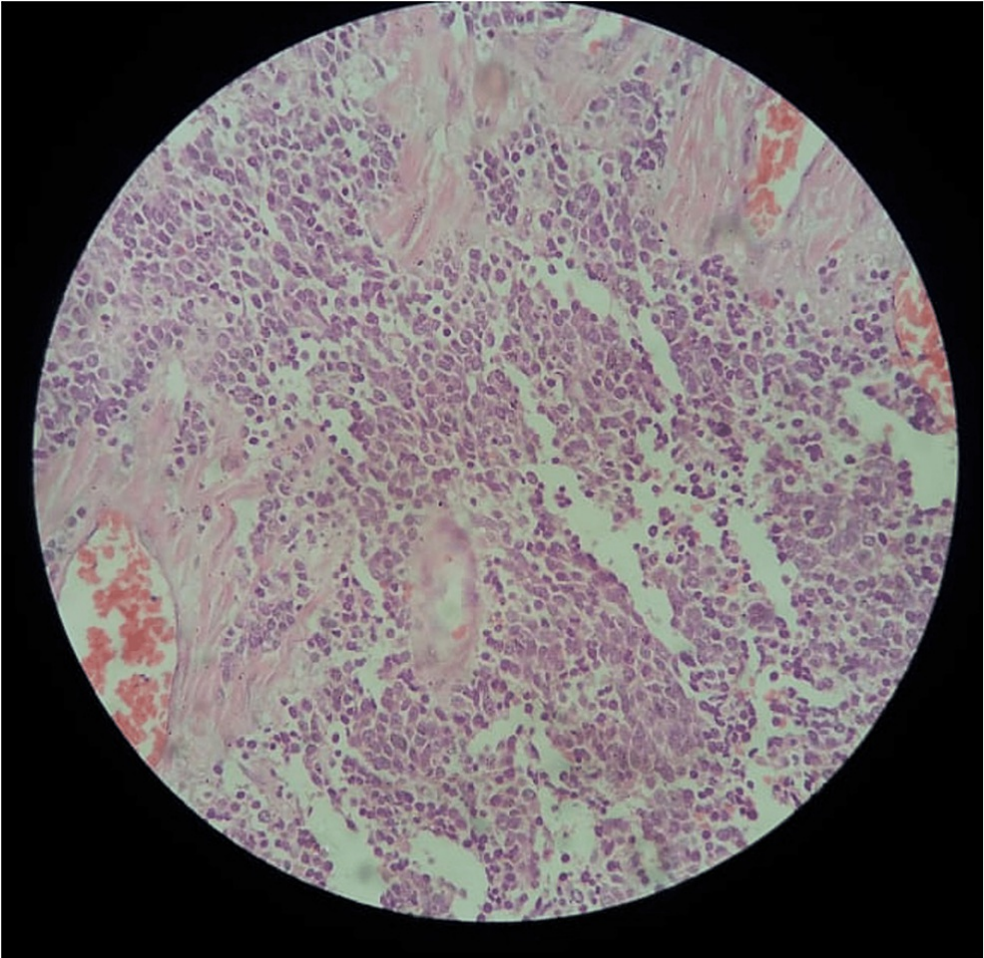
Low Power Microscopy of Neuroendocrine Tumor Favoring Small Cell Carcinoma of the Esophagus

Contrary to the expected pathology, the tumor in the upper third was a NET favoring small cell carcinoma (Figure 2) 5 x 3.5 x 2.3 cm in dimensions, confirmed with positive staining for chromogranin and C56 immunostain. While the CRM was free of tumor, lymphovascular and perineural invasion were identified.

Seven of 19 lymph nodes harvested showed metastatic NET with perinodal extension classifying this as Stage pT3N1bMx. The patient underwent adjuvant chemoradiation and remained disease free at four years, after which he was lost to follow up.

## Discussion

Esophageal carcinoma is the eighth most common cancer and the sixth most common cause of death due to cancer in the world [6]. Patients with esophageal cancer frequently have multiple primary cancers, which contributes to poor prognosis and low survival rates [7]. Most reports of synchronous multicentric tumors of the esophagus in the literature show similar histopathology [8]. However, our patient is unique in that he had two separate histopathologic diagnoses.

Double primary malignancies must satisfy the Warren and Gates criteria (Table 1) [2].

**Table 1:** Warren and Gates Criteria for Multiple Primary Malignancies

Warren and Gates Criteria	
	Histological confirmation of malignancy in both the index and secondary tumors
	There should be at least 2 cm of normal mucosa between the tumors
	If the tumors are in the same location, then they should be separated in time by at least five years
	Probability of one being the metastasis of the other must be excluded

Our patient fulfilled these criteria as there was confirmation of two independent malignancies with about 12 cm of normal mucosa between them. The histopathological diagnosis also excluded the possibility of one lesion being a metastasis of the other.

The association between synchronous primary tumors in the aerodigestive tract has been explained by the concept of "field cancerization", according to which the mucous epithelium of the head and neck, lung and esophagus is exposed to common carcinogenic agents, leading to multiple carcinomas in these regions [6]. In our patient, chronic tobacco use served as a carcinogenic agent.

When multiple primary cancers occur with esophageal cancer, the location of the cancers tends to be outside the esophagus. A 1997 study of 202 patients with esophageal cancers showed a 15.3% incidence of primary cancers in other organs, most commonly in the stomach [9]. Similarly, the 2018 study of 538 patients with esophageal cancer showed a 30% incidence of multiple primary cancers, the most common sites being stomach, head and neck, and the colon [6]. Another case report has described the presence of squamous cell carcinoma of the esophagus, adenocarcinoma of the sigmoid colon and hepatocellular carcinoma occurring simultaneously [10]. Among multiple tumors, synchronous squamous cell carcinomas of the esophagus and thyroid cancers have also been described [11]. Unlike what is reported in the literature, both of our patient’s synchronous tumors were located into the esophagus. Additional imaging also ruled out the presence of lesions outside of the esophagus.

Esophageal cancers most often tend to be squamous cell carcinoma or adenocarcinoma and squamous cell carcinomas are most often associated with other primary malignancies [7]. When associated with synchronous primary malignancies, squamous cell carcinomas of the esophagus have been accompanied by histopathological subtypes including adenocarcinomas of the gastrointestinal tract [10, 12-14], signet-ring cell gastric cancers [13], gastric stromal tumors [8], papillary or follicular thyroid cancers [11], or rarely, duodenal carcinoids [15]. Synchronous squamous cell carcinoma of the esophagus and neuroendocrine tumors such as small cell carcinoma in the esophagus or elsewhere, have not been described in the literature so far. This is a unique feature in our patient.

Furthermore, most neuroendocrine tumors, particularly carcinoid tumors, in the esophagus are located at the middle or lower end of the esophagus. They are not aggressive unless associated with features of adenocarcinoma in the same lesion [16, 17]. Our patient’s small cell carcinoma was located in the upper third of the esophagus, an uncommon site. Although not accompanied by adenocarcinoma features, there was a lymphovascular and perineural invasion of the small cell carcinoma, indicating local spread.

Patients with synchronous multiple primary cancers tend to have poorer long-term outcomes after undergoing esophageal resection [6]. Thankfully, our patient responded well to surgery, followed by chemoradiation and remained disease-free for four years until loss to follow-up. we believe that both the small cell carcinoma and squamous cell carcinoma were caught in time before they became aggressive and metastasized distantly.

## Conclusions

While double and triple synchronous primary malignancies have been reported in the literature, these include primary carcinomas, adenocarcinomas, and carcinoid tumors in different parts of the gastrointestinal tract and other solid organs. We report an unusual case of small cell carcinoma and squamous cell carcinoma of the esophagus, that responded well to surgery followed by chemoradiation.
